# Melanocytic cutaneous lesions in *Sus ibericus*, are these a cause for meat condemnation?

**DOI:** 10.17221/5/2024-VETMED

**Published:** 2024-07-31

**Authors:** Jose Castanho, Jose Catarino, Laurentina Pedroso, Antonieta Alvarado, Sonia Ramos

**Affiliations:** ^1^Faculty of Veterinary Medicine, University Lusófona – Lisbon University Center, Campo Grande, Lisbon, Portugal; ^2^School of Animal Health, Protection and Welfare, Lusophone Polytechnic Institute, Lisbon, Portugal; ^3^Veterinary and Animal Research Centre (CECAV), University Lusófona – Lisbon University Center, Campo Grande, Lisbon, Portugal; ^4^Department of Veterinary Medicine, University of Évora, Évora, Portugal; ^5^Centre for Research and Technology of Agro-Environmental and Biological Sciences (CITAB) University of Trás-os-Montes and Alto-Douro, Vila Real, Portugal

**Keywords:** Alentejano pig, meat safety, melanocytic tumours, melanocytoma, melanosis

## Abstract

The Alentejano pig (*Sus ibericus*) is an autochthonous breed of swine from Portugal phylogenetically close to the Iberian breed that is known to develop melanocytic lesions. In this study, 34 melanocytic skin lesions were identified and collected from Alentejano pigs slaughtered for human consumption for further routine histologic assessment. The samples were classified into 4 age ranges: 1 (1 to 6 months), 2 (7 to 12 months) 3 (13 to 24 months), and 4 (more than 25 months). All the lesions were considered benign after the histopathological assessment, of which 52.9% and 47.1%, were classified as melanosis and melanocytomas, respectively. Regarding the age ranges, a statistical difference between the groups was observed, indicating that the probability of melanosis presentation was higher at the age range 4 and for melanocytomas at the age range 3. While no malignant lesions were observed in this study, it was concluded that benign melanocytic lesions are commonly found in Alentejano pig carcasses. Further research is necessary to accurately distinguish between malignant and benign lesions, which is crucial for official veterinarians to make decisions regarding meat approval or condemnation.

Melanocytic cutaneous lesions are occasionally detected in pigs slaughtered for human consumption and the disposal of the meat depends on distinguishing melanosis from melanocytic tumours, accordingly, whether it is benign (melanocytoma) or malignant (melanoma) (Bundza and Feltmate 1990; Teixeira et al. 2013).

Currently, European legislation only states that, whenever pathophysiological changes occur, the meat must be declared unfit for human consumption [Regulation (EU) 2019/627] (European Union 2019) and according to the Codex Alimentarius, the partial rejection of the carcass or viscera should be performed when affected by the presence of localised melanosis or by a benign, single, circumscribed tumour whereas the total rejection when in the presence of generalised melanosis or the presence of malignant melanocytic tumours (FAO 1994).

The Alentejano pig (*Sus ibericus*), Figure 1, constitutes one of the largest populations of Med-iterranean breeds from the Iberian Peninsula, phylogenetically close to the Iberian breed. It is native to rural areas in the Alentejo region, Portugal, and lives freely in the *montado*, an agro-silvo-pastoral system, through the opening and selection of Mediterranean Forest species and their conservation through grazing and agricultural practices (Fabuel et al. 2004; Nunes 2007). This free-living native breed is characterised by slow-growing subjects well adapted to the often, unfavourable environmental conditions, feeding on undergrowth products and fruits (mainly acorn and lande) (Nunes 2007).

**Figure 1:**
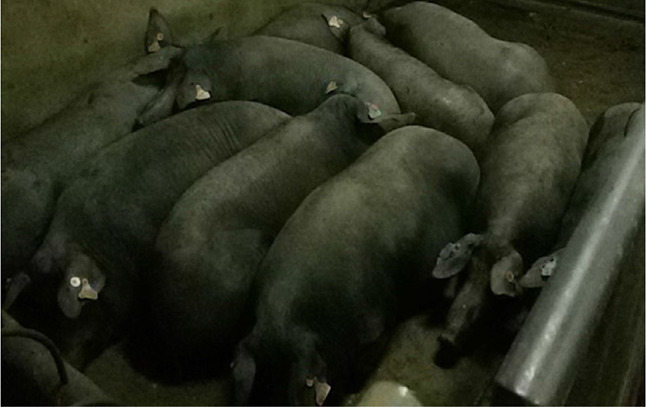
Alentejano pig in the slaughterhouse (original photo)

In Portuguese slaughterhouses, melanocytic lesions have been identified in the Alentejano pig, often leading to condemnation of the meat, however, there are no studies on the prevalence and characterisation of melanocytic lesions in this autochthonous breed. In this study, macroscopic melanocytic cutaneous lesions were identified in the Alentejano pig and collected at the slaughterhouse level for histopathological characterisation, according to their degree of malignancy, to establish a better relationship between their macroscopic observation and the decision by the official veterinarian at the slaughterhouse.

## MATERIAL AND METHODS

For this study, during a 5-month period, 34 melanocytic cutaneous lesions were collected from Alentejano pig carcasses in a slaughterhouse located in the Alentejo region, in Portugal.

To prevent damaging the integrity of carcasses, all the samples were collected at the time of inspection, after the dehairing and singeing process. Additionally, two samples of retropharyngeal lymph nodes with the presence of brown-black pigment were collected from carcasses with melanocytic lesions.

The samples were classified into 4 age ranges: 1 (1 to 6 months), 2 (7 to 12 months), 3 (13 to 24 months) and 4 (more than 25 months). All the collected samples were immediately fixed in 10% buffered formaldehyde. The lesions were macroscopically classified according to Grossi et al. (2015) and processed for routine haematoxylin and eosin staining for histological assessment. Lesions were classified microscopically according to the Histological Classification of Skin Tumours in Domestic Animals proposed by the World Health Organization (WHO) (Goldschmidt et al. 1998). Due to the presence of a large amount of pigment, slides were bleached using potassium permanganate at 0.25% (Perez et al. 2002).

The lesions classified in the group age range were analysed by the probability of the Pearson’s chi-squared test (*P* < 0.05).

## RESULTS

**Figure 2:**
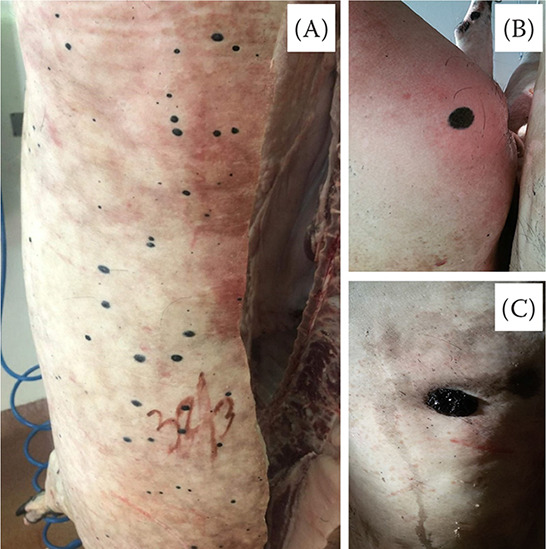
Melanocytic lesions identified on an Alentejano pig’s carcass in the slaughter line, after the dehairing and singeing processes (A,B) It is possible to observe pigmented maculae with up to 5 cm in diameter. (C) Raised black and sometimes ulcerated lesions

Macroscopically, two types of melanocytic lesions were classified as, type-1: pigmented maculae, with a diameter between 0.1 cm to 5 cm and without relief (Figure 2A,B), and type-2: raised and sometimes ulcerated lesions, with a diameter between 0.1 cm and 2.5 cm, discreet edges and an irregular surface (Figure 2C). These lesions showed a brownish-black colour, and pigmentation was mainly observed in the superficial dermis and occasionally in the deep dermis.

**Figure 3:**
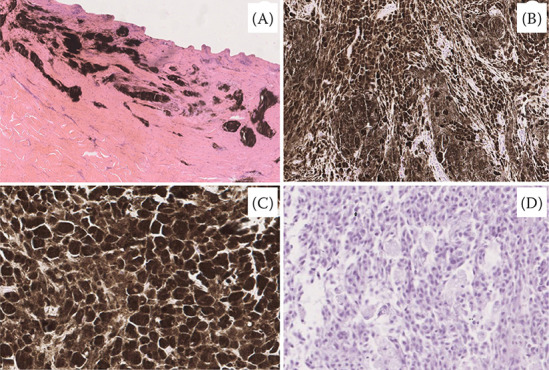
Histological classification of the melanocytic lesions processed (A) Melanosis, observed as a horizontally oriented plaque-like lesion, composed of ovoid to fusiform to polyhedral cells, with abundant cytoplasm laden with black-brown granular pigment corresponding to melanin (haematoxylin and eosin, × 100). (B,C) Melanocytomas, characterised by the proliferation of round to polyhedral cells with abundant cytoplasm obscured by variable-sized pigment intracytoplasmic granules, supported by stromal tissue rich in dense non-patterned collagen (haematoxylin and eosin, × 100 and × 400, respectively). (D) After the bleaching procedure, evidencing nuclei of the pleomorphic melanocytic cells (haematoxylin and eosin, × 100)

Pigmented maculae lesions were microscopically well-demarcated, on the superficial dermis, as a horizontal plaque, composed of ovoid to fusiform cells, some of which were polyhedral, with abundant cytoplasm laden with black-brown granular pigment corresponding to melanin (Figure 3A). Accordingly, 52.9% (18/34) of the collected lesions were identified as melanosis.

On the other hand, all tumour lesions (raised type-2 lesions) were classified as melanocytomas, corresponding to 47.1% (16/34) of the collected lesions. Histologically, these raised lesions were typically well-demarcated, dermal to subcutaneous, composed of round to polyhedral cells, and, in some cases, fusiform, with abundant cytoplasm filled with brown-black granular pigment whose granules varied in size (Figure 3B), with nucleus usually central, sometimes masked by granules of dark pigment (Figure 3C). In some lesions, the cells reached the deep dermis forming a nest of cells, some of which surrounded blood vessels. The stroma was composed of dense non-patterned connective tissue, and some lesions had moderate to abundant lymphocytes and plasma cells in the adjacent stroma. Furthermore, six of these lesions were bleached, and proliferating cell nuclei were evident, most of these were round to ovoid, with dense to euchromatic chromatin and, when visible, between 1 and 2 nucleoli. No mitoses were observed in 10 count fields (obj. 40 × FN 20). Macrophages laden with granular material (blanched granules) and inflammatory cells (lymphocytes, plasma cells, and neutrophils) were observed among the neoplastic cells (Figure 3D).

The two collected retropharyngeal lymph nodes were macroscopically normal, in size and shape, however, showed diffuse black discolouration of the cortex and medulla. Macrophages laden with granular material (brown-black granules), corresponding to melanophages, were observed using haematoxylin and eosin, particularly at the medulla. All the lesions were characterised as benign, suggesting that the diagnosis of malignant melanocytic lesions as melanomas are less frequently detected in slaughterhouses. Regarding the age ranges, there was a significant difference between the groups, indicating that the probability of melanosis was higher at age range 4 (older animals) and at age range 3 (younger animals) for melanocytomas.

## DISCUSSION

During the post-mortem inspection of Alentejano pig carcasses, melanocytic lesions are frequently identified, leading to meat condemnation for human consumption. An extremely high prevalence of melanocytic lesions, with 95% of pigs with cutaneous lesions in Iberian and cross-bred pigs, have been reported (De la Torre et al. 1998). It is known that the Iberian and Alentejano pig breeds have the same genetic origin and are probably connected by a common ancestor (Munoz et al. 2018).

In our study, melanosis was the most prevalent melanocytic cutaneous lesion found (52.9%) and these lesions were characterised as pigmented maculae (with a grey to black colouration) in a focal or multifocal pattern, flat, circumscribed, and not ulcerated, as previously described by others (Jones and Hunt 1983; Teixeira et al. 2013). Melanosis is caused by a migration of melanocytes during embryogenesis and is characterised by an abnormal accumulation of melanin pigment (Lambert et al. 2019), which, in pigs, is mostly found in the lymph nodes, skin, and belly fat or mammary tissue (Herenda et al. 1994; Lanteri et al. 2009). Melanosis is often reported in cattle slaughtered for human consumption and is occasionally described in pigs (Hernandez De Lujan et al. 2009; Lanteri et al. 2009; Morey-Matamalas et al. 2021). On the other hand, it has also been reported as the most frequent melanocytic lesion in pigs at slaughter (De la Torre et al. 1998; Teixeira et al. 2013).

Although, melanosis may have a congenital aetiology in the Sinclair, Duroc, Hormel, and Vietnamese swine breeds (Bundza and Feltmate 1990), in Nero Siciliano pig and Calabrian black pigs, the acquired melanosis has been reported as resulting from an acorn-based diet, based on the hypothesis that this diet is rich in some precursors of melanin pigment (Lanteri et al. 2009; Lanteri et al. 2019). Likewise, we can hypothesise that melanosis in the Alentejo pig may have a genetic origin and/or be associated with the type of diet.

Moreover, congenital melanosis in some swine breeds has been linked to tumours (Teixeira et al. 2013), and Duroc, Sinclair, Hormel, Hampshire, and Iberian breeds have a hereditary predisposition to develop cutaneous melanocytic tumours (Perez et al. 2002; Teixeira et al. 2013). Additionally, a multifactorial aetiology, such as solar radiation and chemical agents, has been described in melanocytic tumours (Smith et al. 2002). Considering the genetic origin and the free-living conditions of the Alentejano pig, we suspect that this is a breed with a predisposition to developing melanocytic tumours.

Although melanocytic tumours are rare in swine, in slaughtered pigs they have been mainly reported in the Duroc and Duroc cross-breeds (Grossi et al. 2015) and, in our study, all the tumour lesions (raised lesions) were classified as melanocytomas. In a previous study, conducted in several Portuguese pig slaughterhouses, melanomas were more frequently reported (21.15%) when compared to melanocytomas (4.81%) (Teixeira et al. 2013), nevertheless, this study was mainly conducted in commercial cross-breeds. In line with our results, a very low percentage (0.15%) of malignant melanomas have been reported in cross-bred Iberian × Duroc pigs and pure-bred Iberian pigs (De la Torre et al. 1998).

As in our study, Grossi et al. (2015) identified pigmented maculae and raised tumours as distinct types of melanocytoma in pigs. Macroscopic characteristics, such as an elevated, rough surface, as well as ulceration, have been suggested as possible indicators of malignancy (Smith et al. 2002; Teixeira et al. 2013), which, in the post-mortem assessment of these tumour lesions, may mislead the decision of the official veterinarian. However, in our study, pigmented raised lesions were classified as melanocytomas, meaning that some criteria must be established to make a correct decision when accepting or rejecting the carcass for human consumption.

Previously, it was proposed that these raised tumours occur when the pigment present in the dermis is phagocytised by macrophages causing an increase in volume and thereby an elevation of the epidermis, moreover, the transition of heavily pigmented maculae to raised black tumours occurs after the progressive regression of the skin lesion (Flatt et al. 1968; Grossi et al. 2015). In pigs, is difficult to differentiate melanophages (macrophages that phagocyte melanin) from melanocytes, but it is possible using an immunohistochemistry technique with ionised calcium-binding adaptor molecule 1 (Iba1) antibodies in the skin and lymph nodes. Another important difference that can be useful for the routine diagnosis is that melanocytic pigment granules in melanophages are coarser than those found in melanocytes (Grossi et al. 2015; Goldschmidt and Goldschmidt 2016).

In all animal species, melanomas share a similar biology in that they frequently recur and are predisposed to metastasise to a regional lymph node (Smith et al. 2002). At the slaughterhouse, during post-mortem inspection, the identification of lesions on the skin and in the regional lymph node can be interpreted by the official veterinarian as a sign of malignancy, leading to meat condemnation. The lesions, observed in the two retropharyngeal lymph nodes collected, were characteristic of melanosis. The spontaneous regression of melanocytic tumours can be characterised by a massive infiltration of pigmented macrophages and lymphocytes and, the black pigmentation of regional lymph nodes has been associated with metastatic growth of pigment-producing malignant melanomas or by pigment-laden macrophages from melanomas undergoing regression (Grossi et al. 2015). As such, regional lymph nodes should be correlated macroscopically and microscopically to issue an accurate diagnosis.

In pigs that develop cutaneous melanocytomas, regression is a common occurrence and may occur *in utero* and at various times after birth. At one year of age, 95% of cutaneous melanomas have undergone or are undergoing regression (Bundza and Feltmate 1990), which could explain why, in this study, the older animals had fewer melanocytomas than the young animals.

Even though, in the present study, we did not observe any malignant lesions, it was possible to conclude that melanocytic lesions in Alentejano pigs are frequently identified in carcasses from pigs slaughtered for human consumption. The characterisation of these melanocytic cutaneous lesions in the Alentejano pig is extremely important, since distinct lesions may reflect different sanitary decisions. All the lesions characterised in this study were classified as benign and did not pose any direct health risk for the consumer. However, as histopathological examinations are laborious and time-consuming, during meat controls at slaughter, it is impossible to identify the neoplastic nature of these lesions or determine their malignancy, leading the official veterinarian to condemn the meat for human consumption, even in the presence of benign lesions.

As far as we know, the characterisation of the melanocytic lesions found in Alentejo pigs could influence the acceptance of the meat by consumers, as they do not represent a public health risk, however, the detection of melanocytic cutaneous lesions in pigs slaughtered for human consumption have a big economic impact, affecting the carcass quality, decreasing the consumer appeal, pork marketability and threatening the sustainable production of this autochthonous Portuguese breed. Further studies on the histological evaluation of these lesions, complemented with immunohistochemistry and oncogenetic studies, should be performed to understand their malignancy.

## References

[R1] Bundza A, Feltmate TE. Melanocytic cutaneous lesions and melanotic regional lymph nodes in slaughter swine. Can J Vet Res. 1990 Apr;54(12):301-4. 2357671 PMC1255655

[R2] De la Torre R, Villamandos JCG, Aparicio JP, Perez MJB, Bienes MH, Sierra MA, Gonzalez-Albo JMM. Hiperpigmentaciones melanicas en cerdos de raza iberica y sus cruces. Prevalencia y tipos [Melanic hyperpigmentation in Iberian pigs and their crosses. Prevalence and types]. Med Vet. 1998 Dec;15(12):668-74. Spanish.

[R3] European Union. Regulation (EU) No. 2019/627 of the Commission, lays down uniform practical arrangements for the performance of official controls on products of animal origin intended for human consumption. OFEU. 2019 Mar. 51-99 p.

[R4] Fabuel E, Barragan C, Silio L, Rodriguez MC, Toro M. Analysis of genetic diversity and conservation priorities in Iberian pigs based on microsatellite markers. Heredity. 2004 May;93(1):104-13. 15150539 10.1038/sj.hdy.6800488

[R5] FAO/WHO – Food and Agriculture Organization of the United Nations/World Health Organization. Codex alimentarius. Vol. 10. Meat and meat products, including soups and broths. 2^nd^ ed. Rome: Food and Agriculture Organization of the United Nations, World Health Organization; 1994. 159-241 p.

[R6] Flatt RE, Middleton CC, Tumbleson ME, Perez-Mesa C. Pathogenesis of benign cutaneous melanomas in miniature swine. J Am Vet Med Assoc. 1968 Oct;153(7):936-41. 5692934

[R7] Goldschmidt MH, Dunstan RW, Stannard AA, von Tscharner C, Walder EJ, Yager JA. Melanocytic tumors and tumor-like lesions. In: Goldschmidt MH, Dunstan RW, Stannard AA, von Tscharner C, Walder EJ, Yager JA, editors. Histological classification of epithelial and melanocytic tumors of the skin of domestic animals. Washington, DC: Armed Forces Institute of Pathology; 1998. 38-40 p.

[R8] Goldschmidt MH, Goldschmidt KH. Epithelial and melanocytic tumors of the skin. In: Meuten DJ, editor. Tumors in domestic animals. Hoboken, NJ, USA: John Wiley and Sons, Inc.; 2016. 88-141 p.

[R9] Grossi AB, Hyttel P, Jensen HE, Leifsson PS. Porcine melanotic cutaneous lesions and lymph nodes: Immunohistochemical differentiation of melanocytes and melanophages. Vet Pathol. 2015 Jan;52(1):83-91. 24503437 10.1177/0300985814521637

[R10] Herenda DC, Chambers PG, Ettriqui A, Seneviratna P, Da Silva TJP. Manual on meat inspection for developing countries [Internet]. 1994 [cited 2023 Aug 28]. Rome: Food and Agriculture Organization of the United Nations. 357 p. Available from: https://www.fao.org/3/t0756e/T0756E02.htm#ch2.12.

[R11] Hernandez De Lujan S, Anton C, Jaro P, Fustel M, Peno J, Carcel MJ. Prevalence of melanosis in cattle slaughtered in Spain. Vet Rec. 2009 Jun;164(23):722-3. 19502629 10.1136/vr.164.23.722

[R12] Jones T, Hunt R. Veterinary pathology. 5^th^ ed. Philadelphia: Lea and Febiger; 1983. 79-82 p.

[R13] Lambert MW, Maddukuri S, Karanfilian KM, Elias ML, Lambert WC. The physiology of melanin deposition in health and disease. Clin Dermatol. 2019 Sep-Oct;37(5):402-17. 31896398 10.1016/j.clindermatol.2019.07.013

[R14] Lanteri G, Marino F, Lagana G, Bellocco E, Barreca D, Liotta L, Sfacteria A, Macri B. Acquired melanosis caused by acorn ingestion in the Nero Siciliano pig. Vet Pathol. 2009 Mar;46(2):329-33. 19261647 10.1354/vp.46-2-329

[R15] Lanteri G, Abbate JM, Iaria C, Macri D, Ferrantelli V, Marino F. Acorn-related acquired pseudomelanosis in Ca-labrian black pigs. BMC Vet Res. 2019 Jun;15(1):186. 31164162 10.1186/s12917-019-1934-5 PMC6549356

[R16] Morey-Matamalas A, Vidal E, Martinez J, Alomar J, Ramis A, Marco A, Domingo M, Segales J. Neoplastic lesions in domestic pigs detected at slaughter: Literature review and a 20-year review (1998–2018) of carcass inspection in Catalonia. Porcine Health Manag. 2021 Apr;7(1):30. 33827694 10.1186/s40813-021-00207-0 PMC8025367

[R17] Munoz M, Bozzi R, Garcia F, Nunez Y, Geraci C, Crovetti A, Garcia-Casco J, Alves E, Skrlep M, Charneca R, Martins JM, Quintanilla R, Tibau J, Kusec G, Djurkin-Kusec I, Mercat MJ, Riquet J, Estelle J, Zimmer C, Razmaite V, Araujo JP, Radovic C, Savic R, Karolyi D, Gallo M, Candek-Potokar M, Fontanesi L, Fernandez AI, Ovilo C. Diversity across major and candidate genes in European local pig breeds. PLoS One. 2018 Nov 20;13(11):e0207475. 30458028 10.1371/journal.pone.0207475 PMC6245784

[R18] Nunes J. Producao pecuaria no montado: Suinos [Livestock production in the “montado”: Pigs]. Rev de Cienc Agrarias. 2007 Jan; 30(1):251-9. Portuguese.

[R19] Perez J, Garcia PM, Bautista MJ, Millan Y, Ordas J, Martin De Las Mulas J. Immunohistochemical characterization of tumor cells and inflammatory infiltrate associated with cutaneous melanocytic tumors of Duroc and Iberian swine. Vet Pathol. 2002 Jul;39(4):445-51. 12126147 10.1354/vp.39-4-445

[R20] Smith SH, Goldschmidt MH, McManus PM. A comparative review of melanocytic neoplasms. Vet Pathol. 2002 Nov;39(6):651-78. 12450197 10.1354/vp.39-6-651

[R21] Teixeira C, Pires I, Ferreira S, Vieira-Pinto M. Lesoes melanociticas em suinos abatidos para consumo [Melanocytic lesions in pigs slaughtered for consumption]. Arq Bras Med Vet Zootec. 2013 Jan; 65(3):783-91. Portuguese.

